# A Clustering Algorithm for Multi-Modal Heterogeneous Big Data With Abnormal Data

**DOI:** 10.3389/fnbot.2021.680613

**Published:** 2021-06-14

**Authors:** An Yan, Wei Wang, Yi Ren, HongWei Geng

**Affiliations:** ^1^Xinjiang Agricultural University, Ürümqi, China; ^2^Anyang Institute of Technology, Anyang, China

**Keywords:** multi-view, BP neural network, missing attributes, Kmeans, noise reduction processing, data integrity

## Abstract

The problems of data abnormalities and missing data are puzzling the traditional multi-modal heterogeneous big data clustering. In order to solve this issue, a multi-view heterogeneous big data clustering algorithm based on improved Kmeans clustering is established in this paper. At first, for the big data which involve heterogeneous data, based on multi view data analyzing, we propose an advanced Kmeans algorithm on the base of multi view heterogeneous system to determine the similarity detection metrics. Then, a BP neural network method is used to predict the missing attribute values, complete the missing data and restore the big data structure in heterogeneous state. Last, we ulteriorly propose a data denoising algorithm to denoise the abnormal data. Based on the above methods, we construct a framework namely BPK-means to resolve the problems of data abnormalities and missing data. Our solution approach is evaluated through rigorous performance evaluation study. Compared with the original algorithm, both theoretical verification and experimental results show that the accuracy of the proposed method is greatly improved.

## Introduction

As the carrier of information, data must accurately and reliably reflect the objective things in the real world (Murtagh and Pierre, 2014; Brzezińska and Horyń, 2020). How to extract effective information on a large number of data sets for data mining, in addition to effective data analysis technology, good data quality is the basic condition of various data mining (Adnan et al., 2020). In the era of big data, data quality is a key issue that restricts the development of the data industry (Zeng et al., 2021). Therefore, how to effectively ensure the integrity and accuracy of the data and improve the data quality has become an urgent problem to be solved. As data collection and data expression methods become more and more diversified, it has become more convenient to obtain a large amount of multi-source heterogeneous data (Rashidi et al., 2020; Wu et al., 2020). The emergence of multi-source heterogeneous data and the need to mine the inherent information on such data naturally gave rise to modeling learning for multi-source heterogeneous data. Currently, there are two main forms of multi-source heterogeneous data: multi-modal data and multi-view data. Multi-view data refers to the data obtained by describing the same thing from different ways or different angles (Kaur et al., 2019). The meaning of multi-view includes multi-modality, multi-view can express a wider range of practical problems (Ma X. et al., 2021). At present, data labels are usually difficult to obtain, the manifestation of heterogeneous data itself is extremely different. In addition, the noise and outliers contained in the original data put forward higher requirements on the robustness of the algorithm (Ma et al., 2020; Yang et al., 2020). In particular, there are often more noise and outliers in multi-source heterogeneous data, which greatly affects the performance of the algorithm in practical applications. Therefore, unsupervised learning for multi-source heterogeneous data has important theoretical research value and broader application scenarios (Li et al., 2018).

In multi-view data, the information contained in different views usually complements each other (Sang, 2020). Fully mining the data of each view to obtain more comprehensive information is the main goal of multi-source heterogeneous data learning. The earliest multi-source heterogeneous data learning model can be traced back to the two-source data learning model based on canonical correlation analysis (Ruan et al., 2020), which mines the consistent structure information of the data on the basis of the correlation between the two-source data. In addition, Bickel and Scheffer proposed a k-means-based multi-view clustering algorithm and used it to analyze data with two conditionally independent views for text clustering. Referring to the existing literature, the model proposed by (Bickel and Scheffer, 2004) in 2004 is the first literature to study multi-view clustering. (De Sa, 2005) proposed a simple and effective spectral clustering algorithm in the literature, and used the algorithm to process web page data containing two views. This method first uses the similarity matrix to fuse the feature information of the two views, and then uses the classical spectral clustering algorithm to perform clustering and obtain the final clustering result (Zhou et al., 2020).

Self-organizing map (SOM) is an algorithm that uses artificial neural networks for clustering (Ma J. et al., 2021). This method processes all the sample points one by one, and maps the cluster centers to a two-dimensional space to realize visualization. (Yu et al., 2015) proposed an intuitionistic fuzzy kernel clustering algorithm based on particle swarm optimization. (Wu and Huang, 2015) proposed a new DP-DBS can clustering algorithm based on differential privacy protection, which implements a differential protection mechanism. Deep learning is another leap-forward development of artificial neural networks (Ventura et al., 2019). At present, clustering algorithms based on deep learning have also become a hot topic of research. The above-mentioned neural network-based Kmeans algorithm improves the clustering effect in the optimization process after the clustering center is given, but it does not clearly provide a method to determine the lack of cluster numerical attributes and data abnormalities. At the same time, there are many types and attributes of structured data and data collection is becoming more and more complex, resulting in more and more data shortages and data abnormalities. However, it is not that missing data is worthless and often it may be the value of missing data.

Aiming at the above problems, this paper proposes a BPK-means algorithm to improve the BP neural network. The algorithm first predicts the missing attribute values based on the BP neural network, which greatly improves the integrity and reliability of the data; then demises the abnormal data, and finally clusters the same data through different views to verify the difference relevance to attributes and clustering research.

The rest of the paper is organized as follows: Section Preliminaries discusses the basic algorithm. Section Problem Formalization presents the formally define the BPK-means algorithm. Section Optimization of Kmeans clustering algorithm proposes optimization of Kmeans clustering algorithm namely BPK-means algorithm. Finally, we analyze the proposed algorithm through experimental results in Section Experiment and Result Analysis.

## Preliminaries

In this part, it mainly provides the basic algorithm of Kmeans algorithm and BP neural network, which are studied in this paper.

### Kmeans Algorithm

The traditional Kmeans algorithm is an unsupervised learning algorithm, that is to cluster the unlabeled data set. Algorithm main idea is as follows: firstly, *K* initial cluster centers are randomly selected, and each cluster center represents a data set cluster. Then the data points are divided into the nearest data cluster. Finally, the data cluster center is recalculated until the clustering criterion function converges. The convergence function is defined as follows:

(1) $$\begin{array}{l} E=\overset{k}{\underset{i=1}{\sum }}{\underset{x\in C_{i}}{\sum }\|x-u_{i}\|}_{2}^{2} \end{array}$$

Where *E* is the minimum square error of *C* = {*C*_1_, *C*_2_, …, *C*_*k*_} obtained by K-means algorithm for *D* = {*x*_1_, *x*_2_, …, *x*_*k*_}, and *u*_*i*_ is:

(2) $$\begin{array}{l} u_{i}=\frac{1}{\left|C_{i}\right|}\sum_{x\in C_{i}}x \end{array}$$

Where *u*_*i*_ is the mean vector of the *C*_*i*_. Intuitively, the above formula describes the closeness of the samples in the cluster around the cluster mean vector to a certain extent. Generally, the smaller the *E* is, the higher the similarity of samples in the cluster is.

**Definition 1** Euclidean distance is the linear distance between two points in Euclidean space. The Euclidean distance between sample *x*_*i*_ and *x*_*j*_ in m-dimensional space is as follows:

(3) $$\begin{array}{l} d\left(x_{i},x_{j}\right)=\sqrt{\overset{m}{\underset{k=1}{\sum }}{\left(x_{ik}-x_{jk}\right)}^{2}} \end{array}$$

The original K-means algorithm determines the similarity of samples according to Euclidean distance.

### BP Neural Networks

The more layers of neural network, the stronger learning ability. Generally, the learning ability of multi-layer network is better than that of single-layer network, but multi-layer network needs stronger learning algorithm. BP neural network algorithm is one of them, which is widely used.

BP neural network algorithm is a kind of multilayer feedforward network. Firstly, the difference between the output values and the expected value of the network is calculated. Then the partial derivative of the difference is obtained by using the function derivative method, and the feedback processing is carried out along the opposite direction of signal transmission of the system.

The basic idea of BP neural network learning algorithm is as follows (Hosseini and Azar, 2016; Li et al., 2017; Kanaan-Izquierdo et al., 2018; Wu et al., 2019):firstly, the data are input into the neural network of the selected samples. Then the results are processed and calculated in the hidden layer and the output results are taken as the input signals of the next layer, thus the error between the results of the output layers and the expected value of the neural network is obtained. Finally, the connection weights of the interconnected neurons in the neural network are adjusted to the direction of the minimum value of the error surface, and the error solving process is repeated until the output error of the whole neural network reaches the accuracy required.

**Figure 1:**
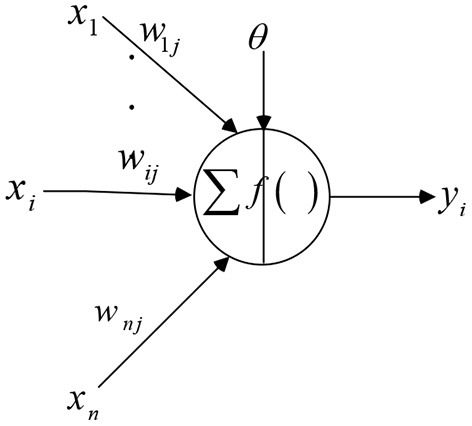
Schematic diagram of BP neuron model.

The learning rule of BP neural network adopts the steepest descent method. Through the back propagation of the network, the weights and thresholds of the network are adjusted continuously to minimize the output error of the network. The topological structure of BP neural network models includes input layer, hidden layer and output layer. The BP neuron model is shown in Figure 1.

Let the input signal of BP neuron be *P*, the weight and threshold be *w* and *b*, respectively, and the processing result be *y*. Transfer functions are commonly used as *logsig* and *tansig*. The formula of *logsig* function is as follows:

(4) $$\begin{array}{l} a=\text{log }sig\left(w+\text{b}\right) \end{array}$$

**Figure 2:**
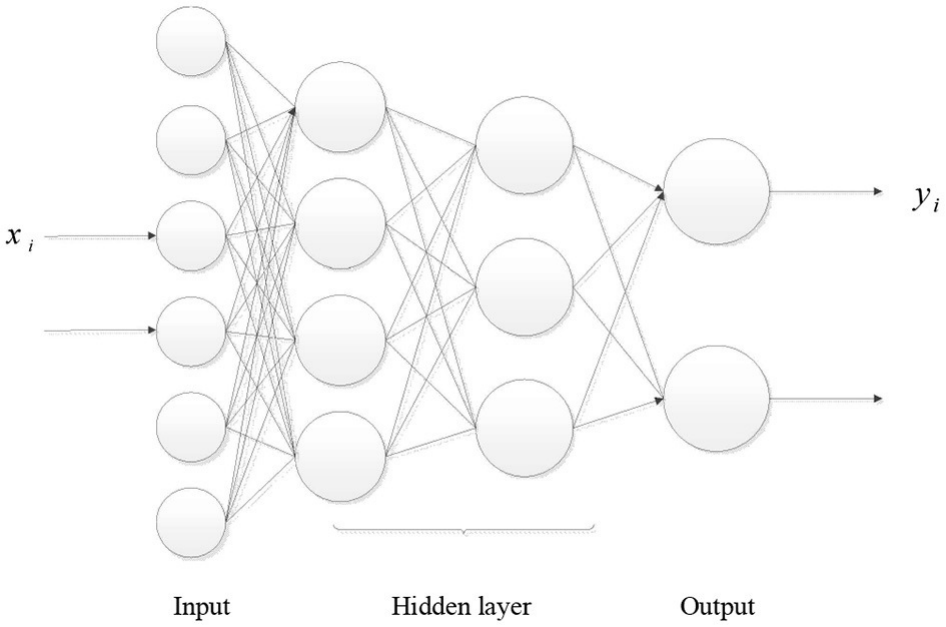
Schematic diagram of BP neural network structure.

The structure of BP neural network is shown in Figure 2, including input layer, hidden layer and output layer.

## Problem Formalization

In this part, we introduce the models for scientific workflows. Then, we formulate the scheduling problem. To improve the readability, we sum up the main notations used throughout this paper in Table 1.

**Table 1:** The main notations.

**Symbols**	**Definitions**
$E=\overset{k}{\underset{i=1}{\sum }}{\underset{x\in C_{i}}{\sum }\|x-u_{i}\|}_{2}^{2}$	Convergence function
*D* = {*x*_1_, *x*_2_, …, *x*_*k*_}	Original data samples
*C* = {*C*_1_, *C*_2_, …, *C*_*k*_}	Clusters
$u_{i}=\frac{1}{\left|C_{i}\right|}\sum_{x\in C_{i}}x$	The mean vector of the *C*_*i*_
$d\left(x_{i},x_{j}\right)=\sqrt{\overset{m}{\underset{k=1}{\sum }}{\left(x_{ik}-x_{jk}\right)}^{2}}$	Euclidean distance
*a* = log*sig*(*w* + *b*)	Transfer function
*p* ∪ *q* = ϕ	itp is missing data, and itq is noisy data
$\begin{array}{l} \delta \left(x,y\right)=\{\begin{array}{c} \;1\;if\left(x=y\right)\\ \;0\;otherwise \end{array} \end{array}$	Represents an indicator function
$ca=\frac{\overset{n}{\underset{i=1}{\sum }}\delta \left(t_{i},map\left(r_{i}\right)\right)}{n}$	Clustering precision
*B* = {*b*_1_, *b*_2_, …, *b*_*n*_}	Data samples after attribute completion
*A* = {*a*_1_, *a*_2_, *a*_3_, …, *a*_*n*_}	Data samples after noise reduction processing
*R* = {*r*_1_, *r*_2_, *r*_3_, …, *r*_*k*_}	The divided clusters of the cluster
{*v*_1_, *v*_2_, *v*_3_, …, *v*_*k*_}	The initial cluster center
$d_{ij}={\left\|x_{j}-v_{i}\right\|}^{2}$	The distance from *x*_*j*_ to each vector *v*_*i*_
*r*_*j*_ = arg min_*i*∈{1, 2, …, *k*}_*d*_*ji*_	Mark the nearest center *x*_*j*_
$f\left(x\right)=\frac{1}{1+e^{-x}}$	The S-type transfer function
$E=\frac{\underset{i}{\sum }{\left(t_{i}+O_{i}\right)}^{2}}{2}$	The error function
$l=\sqrt{m+n}+a$	The number of hidden layers
itTraingdx	The training function
itmse	The performance function
itlr	The learning rate
*I* = (*i*_1_, *i*_2_, …, *i*_*k*_)	Outlier points
$\varepsilon =\frac{\overset{n}{\underset{j=1}{\sum }}|i_{j}-b_{j}|}{n}$	Threshold of outliers error range

### System Models

#### Scientific Model

A model of multi-modal heterogeneous big data with abnormal data in this paper is data aggregation clustering, i.e., *D* = {*x*_1_, *x*_2_, …, *x*_*k*_}. Firstly, we assume that there is no missing and noise data in the initial data *D*, then for the multi-view clustering of *D*, the final clustering accuracy is evaluated according to the combination of different attributes of *D* data. However, for some abnormal data, such as missing and noise data, the clustering effect will be greatly reduced for the same clustering algorithm. Now we assume that for the original data *D*, there are *p* records containing missing values and *q* records containing noise data.

### Problem Formulations

Assuming the data *D*, the attribute field is missing *p* data, and *q* data are noisy data. Suppose *p* ∪ *q* = ϕ, The highest clustering accuracy of the traditional Kmeans algorithm is (*M-p-q*)*/M*. Of course, it's basically impossible.

The goal of our proposed algorithm is that the accuracy is greater than (*M-p-q*)*/M*. In addition, in the multi-modal data view, the traditional Kmeans algorithm has limited fault tolerance for different modal data. The data fault tolerance of our proposed algorithm can be improved. The final result is that the proposed algorithm has a higher clustering accuracy than the Kmeans algorithm. Further, we express it through formal language δ(*x, y*) and *ca* as follows:

(5) $$\begin{array}{l} \delta \left(x,y\right)=\{\begin{array}{c} \;1\;if\left(x=y\right)\\ \;0\;otherwise \end{array} \end{array}$$

(6) $$\begin{array}{l} ca=\frac{\overset{n}{\underset{i=1}{\sum }}\delta \left(t_{i},map\left(r_{i}\right)\right)}{n} \end{array}$$

where *t*_*i*_ is real labels, *r*_*i*_ is labels after clustering, n is the total number of data, δ(*x, y*) represents an indicator function. map function represents the optimal reproduction allocation of class labels.

(7) $$\begin{array}{l} \text{Objective}1:\text{max }ca \end{array}$$

(8) $$\begin{array}{l} \text{Subjectto}:p\cup q=\phi \end{array}$$

## Optimization of Kmeans Clustering Algorithm

**Figure 3:**
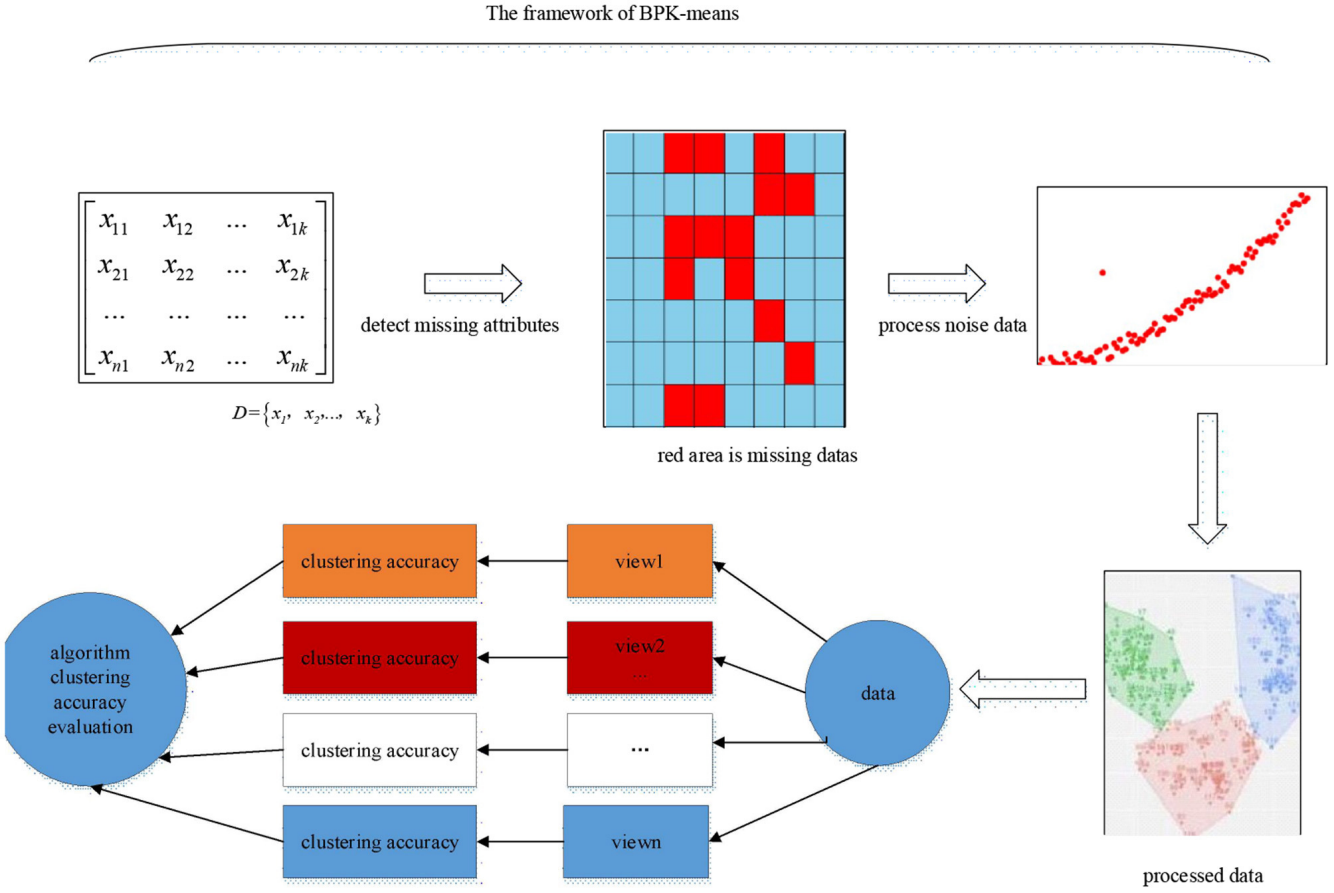
Flow Diagram of BPK-means algorithm.

Generally, for the traditional Kmeans algorithm, the incomplete data is preprocessed and checked, and the preprocessed data is clustered. In addition, a lot of noise data appear due to the variety of acquisition methods. How to fill the missing attribute data and filter the noise data, the traditional Kmeans algorithm does not give a good solution. In view of the above problems, according to the characteristics that BP neural network can well-predict and detect unknown data, this paper proposes a BPK-means algorithm based on BP neural network to improve the Kmeans algorithm. BPK-means algorithm flow chart is described by Figure 3. We use the BP neural network attribute completion algorithm to detect missing attributes and by using Data denoising algorithm to process noise data.

### BPK-Means Algorithm

The improvement of BPK-means algorithm and traditional Kmeans algorithm lies in the optimization of data attribute missing and denoising. Some of the missing attribute data is still meaningful. If it is discarded randomly, the result of data clustering will be inaccurate. BP neural network model can predict the missing data, and the prediction accuracy of the data can reach more than 90%, which greatly ensures the integrity of the data. In addition, outliers are used to analyze some noise data, and then BP neural network is used to judge whether the data is valid. BPK-means algorithm flow is described by Algorithm 1. Besides this, We define several functions in Algorithm 1.

(9) $$\begin{array}{l} d_{ij}\left(x_{j},v_{i}\right)={\left\|x_{j}-v_{i}\right\|}^{2} \end{array}$$

Where *d*_*ij*_(*x*_*j*_, *v*_*i*_) is the distance of *x*_*j*_ and *v*_*i*_.

(10) $$\begin{array}{l} r_{j}=\arg\min_{i\in \left\{1,2,\ldots ,k\right\}}{d_{i}}_{j} \end{array}$$

*r*_*j*_ is the minimum distance of *d*_*ij*_(*x*_*j*_, *v*_*i*_),which can determines cluster markers of *x*_*j*_.

**Algorithm 1:** BPK-means algorithm.

**Input:** Sample set *D* = {*x*_1_, *x*_2_, …, *x*_*k*_}; Number of clusters *k*;
**Output:** The divided clusters of the cluster *R* = {*r*_1_, *r*_2_, *r*_3_, …, *r*_*k*_};
1:Use BP neural network to complete the missing attributes of data set *D*;
2: Using outliers and BP neural network to denoise the data, the processed data set sample is: $$\begin{array}{l} \text{A=}\left\{{\text{a}}_{1}\text{,}{\text{a}}_{2}\text{,}{\text{a}}_{3}\text{,}\ldots \text{,}{\text{a}}_{n}\right\}; \end{array}$$
3: Randomly select k samples from A as the initial vector, that is, the initial cluster center is recorded as the vector: $$\begin{array}{l} \left\{v_{1},v_{2},v_{3},\ldots ,v_{k}\right\}; \end{array}$$
4: Order *C*_*i*_ = ∅(1 ≤ *i* ≤ *k*);
5: Loop *j* = 1, 2, …, *n*;
6: Calculate *a*_*j*_the distance to each vector *v*_*i*_(1 ≤ *i* ≤ *k*) and record it as*d*_*ij*_(*x*_*j*_, *v*_*i*_);
7: The cluster mark determined according to the nearest center *x*_*j*_ point *r*_*j*_;
8: Group the samples *x*_*j*_ into corresponding clusters: $$\begin{array}{l} C_{r_{j}}=C_{r_{j}}\cup x_{j}; \end{array}$$
9: Circulation order *i* = 1, 2, 3, …, *k*;
10: Calculate the new cluster vector${v_{i}}^{\prime }$;
11: If${v_{i}}^{\prime }\neq v_{i}$, at this time, it is necessary to update the cluster class vector to${v_{i}}^{\prime }$;
12: Otherwise, keep the current cluster vector*v*_*i*_ unchanged;
13: End the loop until the cluster vector is not changed.

The BPK-means algorithm adds the integrity recovery of the data set and the detection of noise, which cannot guarantee the integrity of the data, and prevents the loss of important attributes of the data and low clustering accuracy. In order to show the execution process of the BPK-means algorithm more intuitively.

### BP Neural Network Attribute Completion Algorithm

In BPK-means algorithm, BP neural network is used to complete the missing attributes of data set *D* in the first step. Next, the implementation process of the algorithm is described in detail. BP neural network attribute completion algorithm flow is described by Algorithm 2. What's more, We have made some functions to illustrate the application in Algorithm 2.

(11) $$\begin{array}{l} f\left(x\right)\text{=}\frac{1}{1+e^{-x}} \end{array}$$

The function *f*(*x*) is the S-type transfer function, we use it in BP net.

(12) $$\begin{array}{l} E=\frac{\underset{i}{\sum }{\left(t_{i}+O_{i}\right)}^{2}}{2} \end{array}$$

Where *E* is the error function. *t*_*i*_is the expected output and *O*_*i*_ is the computational output of the network.

(13) $$\begin{array}{l} l=\sqrt{m+n}+a \end{array}$$

*l* is the number of hidden layers, Where m is the number of neurons in the input layer, *n* is the number of neurons in the output layer, and a is a constant between (Murtagh and Pierre, 2014; Ma et al., 2020). According to a large number of experimental data, this algorithm sets *a*=*3*.

**Algorithm 2:** BP neural network attribute completion algorithm.

**Input:** sample set *D* = {*x*_1_, *x*_2_, …, *x*_*k*_};
**Output:** the whole data set *B* = {*b*_1_, *b*_2_, …, *b*_*n*_};
1: Scan the data set once. Find out the record number of the data set and record the data set with incomplete attributes as *Q* = {*q*_1_, *q*_2_, *q*_3_, …, *q*_*m*_};
2: Judge the size of *N*. If *N* is more than 100000 records, then randomly select 20% as the training sample of neural network. If *N* is ≤100,000 records, then select 60% of the data set as the training sample set;
3: Three layers BP neural network model is constructed, which are input layer, hidden layer and output layer;
4: The S type transfer function is set *f*(*x*).
5: The inverse error output is set, and the network weight and threshold are adjusted continuously to minimize the error function *E*. According to all the samples selected in the second step, the network is modeled. In this model, the attribute of data set is used as input, and the number of output nodes is set to 1, the *l* is used in the design of hidden layer.
6: The excitation function of hidden layer and output layer is *Tansig* and *Logsig*, respectively. The training function is *Traingdx*. The performance function is *mse*. The number of iterations is 50,000. The expected error goal is 0.000000001, and the learning rate *lr* is 0.01.
7: According to the setting of the network model in the previous steps, the network model is constructed and trained. In this way, the missing data set in *Q* = {*q*_1_, *q*_2_, *q*_3_, …, *q*_*m*_} is predicted, and a complete data set is constructed and recorded as *B* = {*b*_1_, *b*_2_, …, *b*_*n*_}.

BP neural network attribute completion algorithm makes use of the characteristics of BP neural network. It uses effective data to build sample set for model training, and makes prediction and evaluation for missing attribute data. It not only ensures the integrity of the data, but also the artificial data is more scientific and accurate to a certain extent.

### Data Denoising Algorithm

BP neural network is used to denoise data set *D* in the second step. Next, in the third part, the implementation process of the algorithm is described in detail. Data denoising algorithm is described by Algorithm 3. We define the error range function ε in Algorithm 3.

(14) $$\begin{array}{l} \varepsilon \text{=}\frac{\overset{n}{\underset{j=1}{\sum }}|i_{j}-b_{j}|}{n} \end{array}$$

Data denoising can keep the smoothness of data. In this way, the data can be directly clustered after processing to improve the clustering accuracy of the data.

**Algorithm 3:** Data denoising algorithm.

**Input:** Complete sample set *B* = {*b*_1_, *b*_2_, …, *b*_*n*_};
**Output:** Data set after noise reduction *A* = {*a*_1_, *a*_2_, *a*_3_, …, *a*_*n*_};
1: *B* = {*b*_1_, *b*_2_, …, *b*_*n*_}, the data are clustered by initial algorithm using Kmeans algorithm;
2: Finding out the points outside the cluster set. It is called outlier points *I* = (*i*_1_, *i*_2_, …, *i*_*k*_);
3: For each outlier, BP neural network is used to predict the corresponding attribute value, which is compared with the existing values. We define an error range functionε. If ε is greater than the given threshold, it is considered as a noise point for noise processing. Finally, a noise free data set is formed: $$\begin{array}{l} A=\left\{a_{1},a_{2},a_{3},\ldots ,a_{n}\right\}. \end{array}$$

### BPK-Means Algorithm Performance Analysis

The time complexity of traditional Kmeans algorithm depends on the number of attributes per record, the size of data, the number of iterations and the type of clustering. Its time complexity is expressed as O (I^*^n^*^k ^*^m), where *I* is the number of iterations, *k* is the type of clustering, *m* is the scale of data volume, and *n* is the number of attributes of each record. The BPK-means algorithm adds missing attribute field completion and attributes data prediction on the basis of Kmeans algorithm.

Suppose that p data are missing in attribute field and q data are noise data, then (*p* + *q*) neural network data processing is needed. At present, BP neural network has three layers. Assuming that the number of neurons in each layer is *n1, n2*, and *n3*, respectively. For example, if a sample (*n1* ^*^1) carries out feedforward calculation, it needs to carry out two matrix operations and two matrix multiplication. n1^*^n2 and n2^*^n3 calculations are performed respectively. The number of nodes (*n1* and *n3*) in input layer and final output layer is determined and can be regarded as constant. The hidden layer *n2* in the middle can be set by oneself. The time complexity of feedforward computation for a sample should be O(n1^*^n2 + n2^*^n3) = O(*n2*). The time complexity of back propagation is the same as that of feedforward. Suppose there are m training samples in total, and each sample is trained only once, then the time complexity of training a neural network should be O(m^*^n2). Similarly, if a sample is predicted, the time complexity should be O(*n2*).

The total time of calculating each data result is (*m*+*2*) O (*n*_2_). Since the training samples *m* and *n*_2_ can be determined basically, the time of each neural network is also linear. Finally, the total time complexity of BP neural network is(*p*+*q*)(*m*+*2*)O(*n*_2_). The BPK-means algorithm focuses more on the accuracy of the algorithm, so that the time complexity is slightly higher than the original algorithm. For some scenes with high accuracy requirements, the cost is worth it.

## Experiment and Result Analysis

### Experimental Setup and Experimental Environment

In order to verify the effectiveness of the algorithm, four groups of experiments are carried out.

Experiment 1: verify the missing attribute completion algorithm through UCI data set to verify the approximation analysis of missing data and simulation prediction data, and verify the effectiveness of the algorithm.
Experiment 2: test the denoising algorithm in this paper through UCI data set to verify the denoising effect of this algorithm.
Experiment 3: the clustering accuracy of BPK-means algorithm and traditional K-means algorithm is compared and analyzed through different data sets.
Experiment 4: the clustering accuracy of BPK-means algorithm and traditional K-means algorithm is compared and analyzed through different multiple views.

The running environment of the experiment is Windows 7 operating system, 4 GB physical memory, CPU speed of 3.10 GHZ and programming language is Python3.8.

### Experiment and Result Analysis

#### A Completion Algorithm for Verifying Missing Attributes

Iris data (UCI Iris, 2021) set of UCI is selected as the sample data set of the experiment. The data contains four attributes and 150 records. Randomly select 100 records as the training sample set, and then the rest of the data set, randomly select 10, making some attributes missing for the experiment. Then randomly select 10 items in the remaining data set to make some attributes missing for experiment.

**Figure 4:**
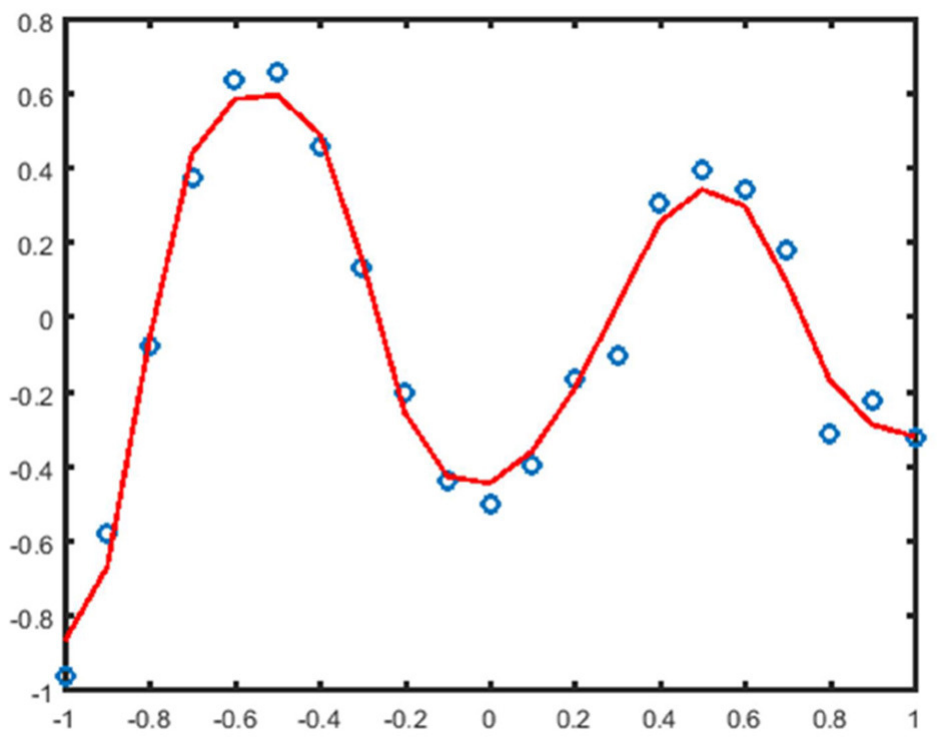
Approximation analysis graph of missing sample attributes.

The above experiments compare the approximation analysis of the predicted value and the real value of the missing attribute data. From Figure 4, the BP neural network can be used to predict the missing attribute data well and achieve the effect of data completion.

#### Validation of Data Denoising Algorithm

Select the complete data set in Experiment 1, and then add noise artificially to verify whether the denoising algorithm is feasible.

**Figure 5:**
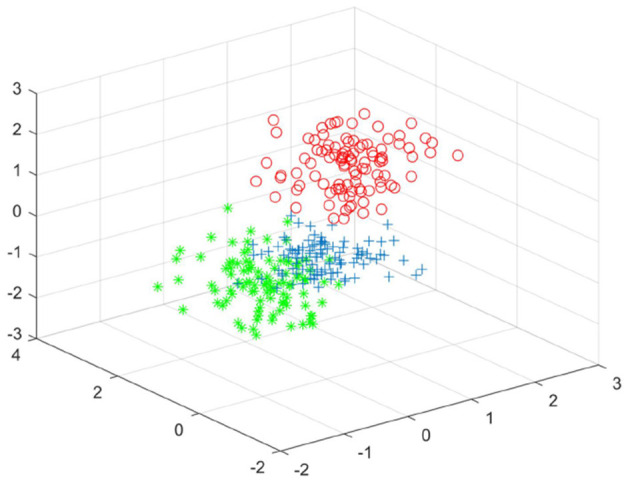
The clustering effect diagram of the completion data of experiment one.

**Figure 6:**
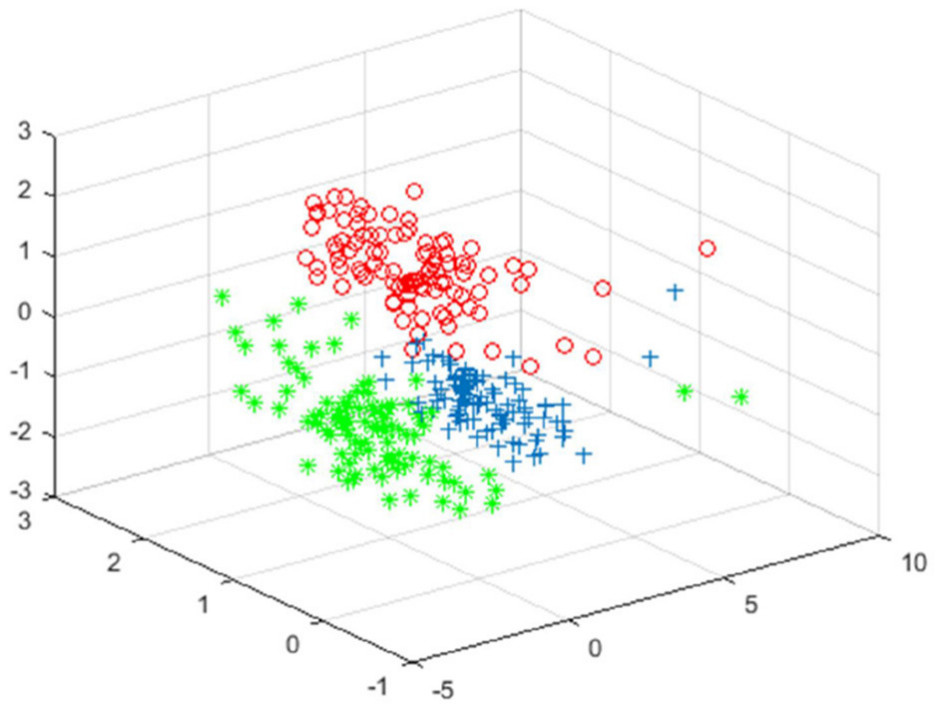
Clustering effect map of the data after noise.

**Figure 7:**
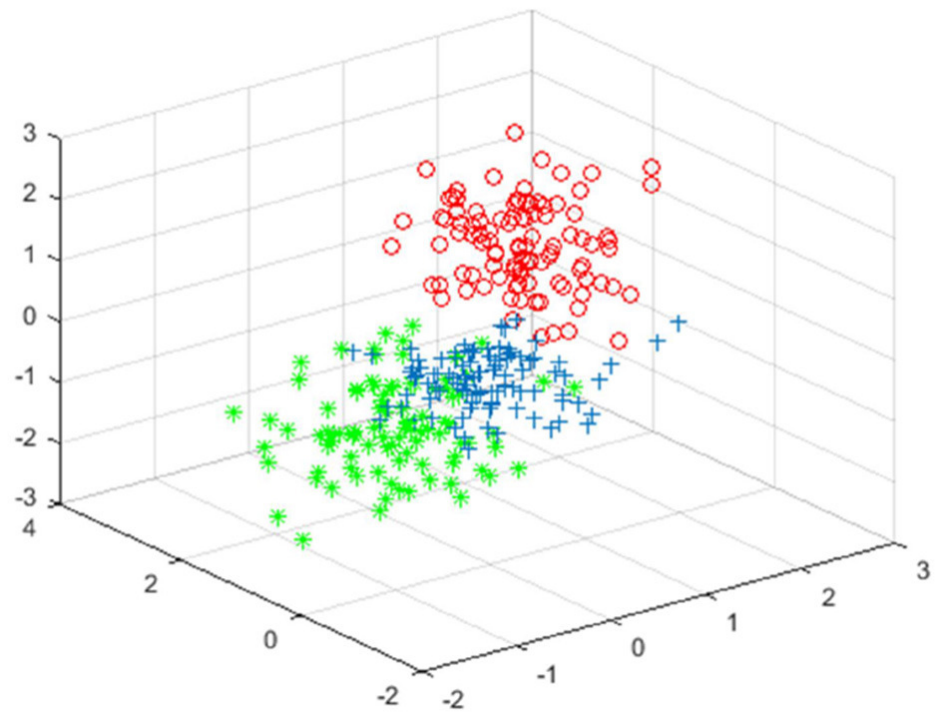
The clustering effect map of the data after noise removal.

Figures 5–7 shows the effect drawing of data completion, the effect picture after noise addition and noise removal, respectively.

#### Validation of BPK-Means Algorithm

The standard data set is selected for clustering verification experiment. The data set is from the public UCI data (UCI Iris, 2021) set. The selected data set randomly set 3–10% missing data attributes to verify the clustering effect of BPK-means algorithm and K-means algorithm.

**Figure 8:**
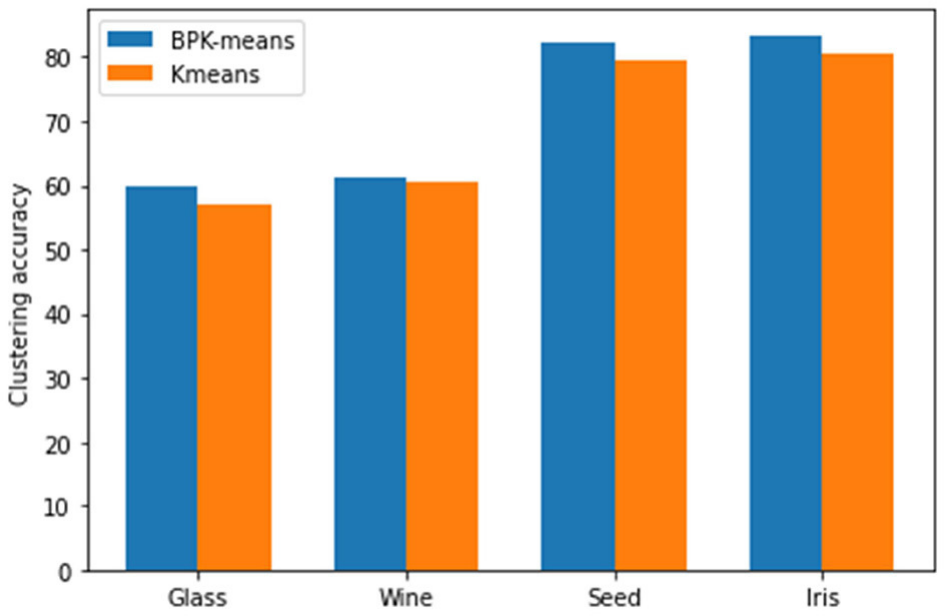
Comparison chart of clustering effect of different data sets.

**Figure 9:**
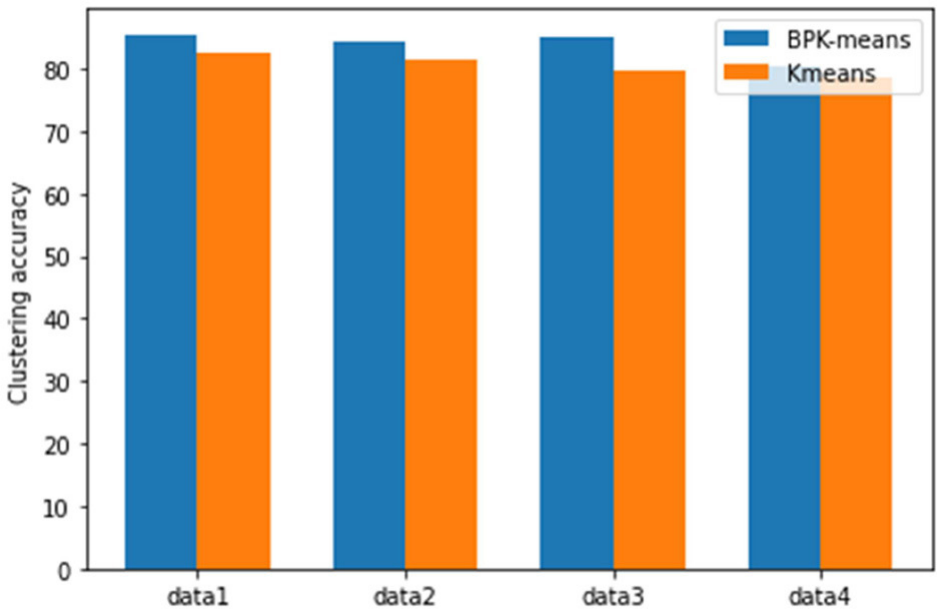
View clustering effect diagram.

Through the comparison of clustering effect of different algorithms and the analysis of UCI data set clustering results from Figure 8, we can see that it is verified that this algorithm has the advantage of high clustering accuracy in the process of clustering analysis for a small number of data with missing attributes about Figure 9.

#### Validation the BPK-Means Algorithm in Multiple Views

The IRIS data set is selected as the experimental data set. The IRIS data set contains 4 columns of data sets and 1 column of label data. The data is randomly added with noise and missing. Sepal length and petal width are selected as data1, petal length and petal width are data2, sepal length and petal length are data3, and sepal width and petal width are data 4 for the accuracy of the multi-view verification algorithm.

By comparing the BPK-means algorithm under different views in Figure 9, the accuracy of the algorithm is improved in different modes, which further shows that the BPK-means algorithm proposed in this paper can be more accurate for some multi-view clustering problems than traditional algorithms.

Through the above four experiments, the traditional algorithm clustering accuracy is performed from attribute missing, data noise, BPK-means algorithm and Kmeans algorithm, and the clustering accuracy of BPK-means algorithm and Kmeans algorithm is verified against Algorithm to verify.

## Conclusion

This paper proposes an improved Kmeans algorithm of BP neural network. The algorithm uses the characteristics of BP neural network to predict and analyze the missing data attribute values. After the completion of the data set, perform data noise reduction processing to remove suspected noise Points are eliminated using BP neural network to ensure the smoothness of the clustering effect, so that the problem of missing data attribute values due to inconsistencies in the form and type of collected data can be solved. Four sets of experiments verify that the BPK-means algorithm proposed in this article can solve the problem of missing attributes, and can solve the clustering accuracy of the data, especially in the multi-view scenario, which is more accurate than the traditional algorithm. However, when the algorithm proposed in this paper is used to process larger data sets, a problem of high time complexity will be generated. Therefore, it is necessary to study how to better improve the accuracy while reducing the time complexity. At the same time dealing with missing data and noisy data reasonably and effectively is also the future improvement and research direction of the algorithm in this paper. This is also the future improvement and research direction of the algorithm in this paper.

## Data Availability Statement

Publicly available datasets were analyzed in this study. This data can be found at: http://archive.ics.uci.edu/ml/datasets/Iris.

## Author Contributions

WW and AY designed the research, performed the experiments, analyzed the data, and wrote the manuscript. YR gave a lot of suggestions in the design of the experiment and the modification of the article. HG performed the experiments. All authors contributed to the article and approved the submitted version.

## Conflict of Interest

The authors declare that the research was conducted in the absence of any commercial or financial relationships that could be construed as a potential conflict of interest.
